# MicroRNAs Detected in Whole Blood Following Traumatic Brain Injury Are Associated with Recovery 6 Months after Injury

**DOI:** 10.1177/2689288X251380526

**Published:** 2025-09-26

**Authors:** Rogan Magee, Junchao Shen, Alexa E. Walter, Andrea L.C. Schneider, Ava M. Puccio, Cillian E. Lynch, Ramon Diaz-Arrastia, Danielle K. Sandsmark

**Affiliations:** ^1^ Department of Neurology, Perelman School of Medicine, University of Pennsylvania, Philadelphia, Pennsylvania, USA.; ^2^ Department of Biostatistics, Epidemiology, and Informatics, Perelman School of Medicine, University of Pennsylvania, Philadelphia, Pennsylvania, USA.; ^3^ Department of Neurosurgery, University of Pittsburgh, Pittsburgh, Pennsylvania, USA.

**Keywords:** biomarkers, microRNA, traumatic brain injury

## Abstract

Traumatic brain injury (TBI) is a heterogeneous disease from which incomplete recovery is unfortunately common. Biomarkers prognostic of future recovery are currently lacking. MicroRNAs (miRNAs), small noncoding RNAs, are attractive potential biomarkers as they are stable in biofluids and can provide molecular mechanistic insights via the mRNAs they regulate. We measured miRNAs from whole blood in TBI participants (*n* = 106) within 24 h of injury and age- and sex-matched control participants (*n* = 107) using the NanoString Technology nCounter® miRNA expression panel. One hundred and nineteen miRNAs were differentially expressed in individuals who had sustained a TBI compared with control participants. Forty-seven of these had levels at 6 months postinjury that were similar to those within 24 h of injury, indicating that these acute differences persisted in the individuals with TBI. Furthermore, two of these 47 miRNAs were upregulated in participants who had incomplete recovery (functional outcome Glasgow Outcome Scale-Extended [GOS-E] score < 8) at 6 months after injury, compared with participants who had complete recoveries (GOS-E = 8). More work is needed to determine if miRNAs may serve as prognostic biomarkers of TBI outcome. MiRNAs may provide insights of the molecular networks and pathophysiological processes impacting recovery after injury warranting future study.

## Introduction

Traumatic brain injuries (TBI) are heterogenous insults. Each injury impacts distinct brain regions, structures, and cell types, as well as having effects on distant organ systems.^1–3^ In addition to the heterogeneity of the initial injury, recovery trajectories after TBI are equally variable. As a result of this complexity, prognostication about the extent of recovery after TBI for individual patients is difficult. Up to 30% of patients with mild TBI/concussion, as determined using clinical grading systems,^4^ report incomplete recovery 6 months after injury.^5,6^ The ability to identify those at-risk individuals in the period immediately following their injury is severely lacking. This limitation makes it difficult to identify patients who need referral to rehabilitation services and remains a barrier to the development of effective neuroprotective and neurorestorative therapies.^7^ As a result, predictors of poor outcomes remain a major unmet research need. Toward this end, ongoing efforts have focused on developing biomarkers that can (1) inform patterns of clinical recovery, (2) identify participants at risk of long-term complications, and (3) aid in the identification of therapeutic targets associated with specific disease endophenotypes.

Numerous studies have explored candidate biomarkers that can be utilized for one or more of these purposes, including those based on neuroimaging, electrophysiology, and protein biomarkers, yet these are not routinely used in clinical settings.^8^ Bazarian et al.^9^ demonstrated that glial fibrillary acidic protein (GFAP) and ubiquitin C-terminal hydrolase-L1 (UCH-L1), proteins derived from injured glia and neurons, respectively, could be used to identify participants with computed tomography (CT)-detected traumatic intracranial injury. These findings contributed to FDA clearance of these biomarkers for clinical use.^9^ When used to assess long-term outcomes, GFAP and UCH-L1 levels were helpful for predicting death and severe disability at 6 months after injury, but had more limited utility for predicting incomplete recovery 6 months after injury.^5,10^ GFAP and UCH-L1 proteins are expressed diffusely within the central nervous system, and are not specific to a particular neuropathology. The neuropathology of TBI is complex and involves axonal shearing, vascular injury, hemorrhage, inflammatory responses from CNS-resident macrophages/microglia and from systemic immune regulators, cerebral edema, and other sequelae. Furthermore, the location and extent of these insults are unique for each patient. While changes in circulating levels of these proteins may reflect diffuse injury, their levels may confer little mechanistic insight into the cells impacted or the molecular signaling cascades that ultimately lead to poor outcomes, particularly in milder head injuries. Furthermore, they do not currently play a role in the stratification of patients who may benefit from neuroprotective or neurorestorative treatments that could impact recovery. The NIH-NINDS TBI Classification and Nomenclature Initiative acknowledges these limitations and has recently suggested a new framework that incorporates four pillars (clinical presentation, blood-based biomarkers, neuroimaging, and modifier features) to improve the classification of TBI.^11^ While the initial guidance has focused on available biomarkers such as GFAP and UCH-L1, the expectation is that more precise blood and neuroimaging biomarkers will be incorporated into this framework as markers are validated.

Toward that goal, microRNAs (miRNAs) are emerging blood biomarkers that may provide further information about the underlying pathophysiology contributing to poor outcomes after injury. miRNAs are short noncoding RNAs (18–22 nucleotides) that modulate protein expression by degrading messenger RNAs (mRNAs) or antagonizing their translation.^12,13^ Unique miRNAs target multiple mRNAs via a “seed” sequence.^14^ As a result, individual miRNAs targeting a wide array of mRNAs can act to fine-tune gene expression and alter cellular function at a network level, thereby operating as potent posttranscriptional regulators.^15^ miRNAs are secreted in biofluids, can be readily measured in blood, and are relatively stable and resistant to exonuclease and endonuclease activity, given the modifications that arise during the maturation process.^16^ The accessibility and stability of miRNAs make them attractive candidate biomarkers for inclusion within the emerging CBI-M framework.

In this study, we measured circulating miRNAs in whole blood collected from participants who sustained a recent TBI (<24 h from injury) and compared their profiles to age- and sex-matched control participants. We examined the associations between these miRNAs and functional outcomes at 6 months. By examining changes in circulating miRNA following TBI in this discovery cohort, we explored whether miRNAs may serve as candidate prognostic biomarkers for TBI and generate hypotheses around specific miRNAs to examine through validation studies.

## Methods

### Participants

TBI participants were enrolled in ongoing prospective, observational biorepository studies at the University of Pennsylvania. Individuals between 18 and 65 years old who experienced head trauma and underwent brain CT as part of their clinical evaluation were eligible for participation. Control participants were healthy, nonhospitalized participants or participants who presented to the University of Pennsylvania Emergency Department with a non-head trauma (i.e., minor orthopedic injury only). The prospective studies and secondary analyses were approved by the University of Pennsylvania Institutional Review Board. All participants or their legally authorized representatives provided informed consent for study participation.

Demographics, clinical information, and injury details were collected at the time of enrollment. For TBI participants, biosamples were collected within 24 h of injury. For control participants, a single biosample was collected. For TBI participants, follow up was done at 6 months postinjury, and a blood draw and functional outcome assessment, including the Glasgow Coma Scale-Extended (GOS-E), were performed. Outcomes were dichotomized as complete recovery (GOS-E = 8) or incomplete recovery (GOS-E < 8).

Given the limitations around recruitment that arose during the COVID-19 pandemic, our local TBI biorepository did not enroll sufficient age-matched, uninjured control participants. To mitigate the potential batch effects, we obtained samples collected as part of the 18-center TRACK-TBI observational study.^6^ To perform the age matching, TBI participants were randomly selected from the University of Pennsylvania cohort and compared with the *n* = 277 participant TRACK-TBI friend control cohort with available whole blood samples. Biosample collection in the University of Pennsylvania and TRACK-TBI studies follows identical workflows involving blood collection, processing, and storage.^5^ We performed 1:1 pair matching by age with exact matching on sex. Age- and sex-matched uninjured control participants were selected from the TRACK-TBI biorepository housed at the University of Pittsburgh. In addition, age- and sex-matched participants with TBI were also selected from the TRACK-TBI cohort to control for center effects.

### RNA processing

For miRNA analysis, whole blood samples were obtained from participants who had sustained a TBI and from control participants. Blood was collected in PAXgene® Blood RNA tubes (BD Biosciences, Franklin Lakes, NJ, USA) within 24 h of injury. RNA was isolated with the PAXgene blood miRNA kit (Qiagen, Germantown, MD, USA) according to the manufacturer’s instructions. RNA concentrations were determined using the NanoDrop® spectrophotometer (Thermo Fisher, Waltham, MA, USA).

### Protein biomarkers

For the TBI participants enrolled at the University of Pennsylvania, we measured protein markers in plasma from those participants with available biospecimens. Whole blood (15 mL) was collected and processed into serum and plasma aliquots by trained clinical staff within 24 h of injury and stored at −80°C. The Quanterix Simoa® HD-X platform (Quanterix Corporation, Lexington, MA) was used to determine concentrations of neurofilament light (NFL), GFAP, total tau (tau), and UCH-L1 using the Neurology4-Plex B kit (Quanterix Corporation, Lexington, MA) according to the manufacturer’s instructions.

### MiRNA quantification

Samples were sequenced through the genomic core at the University of Pennsylvania’s Wistar Institute. miRNAs were assayed using the nCounter® Human v3 miRNA expression panel of 798 miRNAs according to the manufacturer’s instructions (Nanostring Technologies, Seattle, WA, USA). Raw read counts were collected using the nCounter® Digital Analyzer.

### Sequencing batch correction

Batch effects influence the results of computational analysis of RNA sequencing and other high-throughput methods. These effects potentially mask the true biological signal in tests of significance such as differential expression. Raw counts were normalized and corrected for sequencing batch using three published methods, including *Remove Unwanted Variance* (RUVg),^17^ *ComBat-seq,*^18^ and *Limma*.^19^ Principal components analysis (PCA) was used for dimensionality reduction to assess the quality of batch correction using these three methods.

### Differential expression analysis

We used DEseq2^20^ to compare samples from control participants and participants who sustained TBI within 24 h of their injury. The control and injury cohorts were not matched on self-reported race. To improve the generalizability of our findings to other cohorts, we included demographic variables and batch labels in all DEseq2 model designs. Differential expression was assessed as a function of injury (control vs. acute injury) using raw read counts and correcting for batch, age, self-reported race, and sex in the differential expression model. Differential expression was also assessed in a separate DEseq2 model as a function of GOS-E outcome using raw read counts as before and correcting for batch, age, self-reported race, and sex. miRNAs with false discovery rate (FDR)- adjusted *p* value >0.05 were considered not significant. All analyses were performed in R version 4.3.0.

### Protein: miRNA correlation

We used Spearman correlation to compare protein biomarkers against RUVg batch-corrected miRNA semiquantitative values, using only those 119 miRNAs that were differentially expressed in our primary comparison. Spearman ρ and 95% confidence intervals were estimated using bootstrapping per the R *boot* package, using a percentile-based estimate over 1,000 samples. Correlations with FDR-adjusted *p* value <0.05 were plotted in a heatmap demonstrating the strength and direction of correlation.

### Quantitative reverse transcription-polymerase chain reaction

To validate the results of our differential expression analysis, we selected three miRNAs for downstream analysis (selection criteria below). miRNA values were compared against the level of hsa-miR-16-5p as an endogenous control, which was chosen given that it exhibited the lowest mean difference in our nCounter® data among the preferred Thermo Fisher endogenous human controls. Source RNAs from a subset of control (*n* = 16) and acute injury (*n* = 16) samples were reverse transcribed using the TaqMan™ Advanced miRNA cDNA Synthesis Kit (Thermo Fisher, Waltham, MA) according to the manufacturer’s instructions. DNA were quantified according to the manufacturer’s instructions using the Fast Mode on a StepOnePlus™ Real-Time PCR System (Applied Biosystems, Waltham, MA) and using predesigned TaqMan™ Advanced miRNA probes against hsa-miR-16-5p, hsa-miR-223-3p, hsa-miR-29a-3p, hsa-let-7b-5p, and the TaqMan™ Fast Advanced Master Mix (Thermo Fisher, Waltham, MA), according to the manufacturer’s instructions. Samples were run in technical triplicate, and water controls for each unique probe were included in the experiments. Cycle thresholds (Ct) were automatically determined per the StepOnePlus system. Ct values were normalized to the corresponding hsa-miR-16-5p value for each sample. Values were compared using the delta–delta Ct method by first subtracting individual miR-16-5p values from the miRNA of interest, finding the mean minuend, and then subtracting the mean control value from TBI value.^21^

## Results

TBI participants (*n* = 106) were from the University of Pennsylvania (*n* = 83) or the TRACK-TBI (*n* = 23) cohorts. Control participants were from the same cohorts (Penn *n* = 51; TRACK-TBI *n* = 74). Participants were predominantly male and were age-matched (Table 1). Most control participants self-identified as White. TBI participants predominantly self-identified as Black on demographic survey data. The mechanism of injury was primarily motor vehicle crashes (61%) and most presented with mild TBI (GCS 13–15, 82%). Forty-nine (50%) TBI participants had TBI-related abnormalities on clinically obtained head CTs. At 6 months after injury, most participants had complete (GOS-E = 8) or near complete (GOS-E = 6–7) recovery (62%).

**Table 1.** Characteristics of the Cohort

	Control (*n* = 125)	TBI (*n* = 106)	*p* Value
Cohort, *n* (%)			
University of Pennsylvania	51 (41%)	83 (78%)	
TRACK-TBI	74 (59%)	23 (22%)	
Age, Median years (IQR)	28 (23–40)	33 (24–50)	0.07
Sex, male, *n* (%)	89 (71%)	83 (78%)	0.13
Race, *n* (%)			<0.001
Black/African American	32 (26%)	62 (58%)	
White/Caucasian	62 (50%)	39 (37%)	
Other	31 (25%)	5 (5%)	
TBI cause, *n* (%)			
Fall	—	18 (20%)	
MVA	—	55 (61%)	
Other	—	17 (19%)	
Unknown	—	16	
GCS at ED arrival, category, *n* (%)			
Mild (13–15)	—	84 (82%)	
Moderate (9–12)	—	6 (6%)	
Severe (3–8)	—	12 (12%)	
Unknown	—	4	
Trauma-related CT abnormality, *n* (%)			
Yes	—	49 (50%)	
No	—	48 (49%)	
Unknown	—	9 (8%)	
GOS-E, 6 months postinjury, *n* (%)			
1	—	1 (1%)	
2	—	2 (2%)	
3	—	4 (4%)	
4	—	4 (4%)	
5	—	3 (3%)	
6	—	19 (18%)	
7	—	27 (25%)	
8	—	20 (19%)	
Unknown		26 (25)%	
Analysis Batch, *n* (%)			<0.001
Batch 1	29 (27%)	44 (42%)	
Batch 2	15 (14%)	35 (32%)	
Batch 3	63 (59%)	27 (27%)	

CT, computed tomography; GOS-E, Glasgow Outcome Scale-Extended; TBI, traumatic brain injury.

**FIG. 1.**
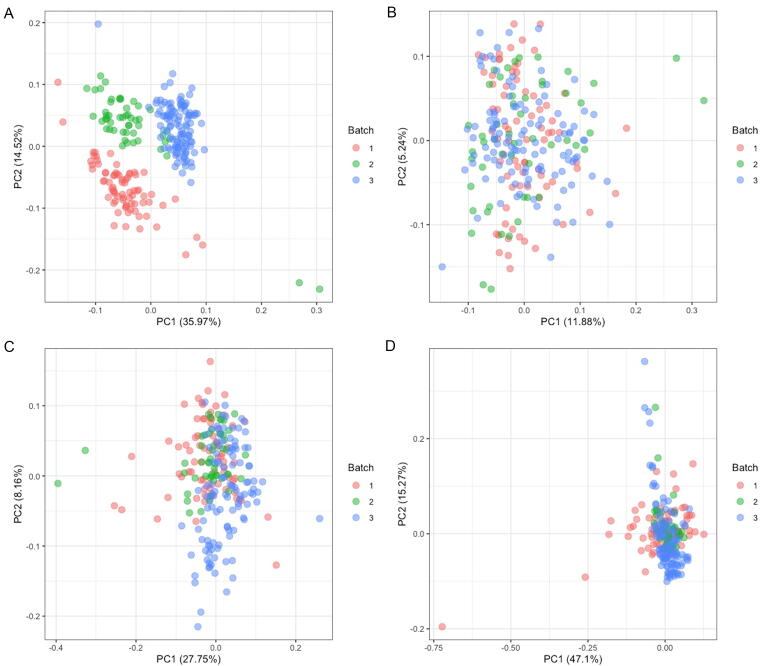
Principal components analysis (PCA) separates samples based on batch effect. PC number 1 (x-axis) and 2 (y-axis) were plotted after PCA was run on raw read data **(A)**, RUVg batch corrected **(B)**, ComBat batch corrected **(C)**, and Limma batch corrected **(D)**. The percentage variance explained by PC1 and PC2 are listed in axis titles. While samples are clearly separated based on batch before correction **(A)**, this effect is mitigated after correction using all three methods **(B–D)**. RUVg batch correction results in the lowest number of clear outliers on the basis of PCA plots **(B)**.

Whole blood samples were analyzed in three batches. PCA of raw nCounter® data demonstrated a significant batch effect within our samples (Fig. 1A). Repeat PCA using three published methods to correct for these batch effects (Fig. 1B–D) demonstrated that the RUVg approach (Fig. 1B) best reduces the influence of sequencing batch on unsupervised sample clustering.

**FIG. 2.**
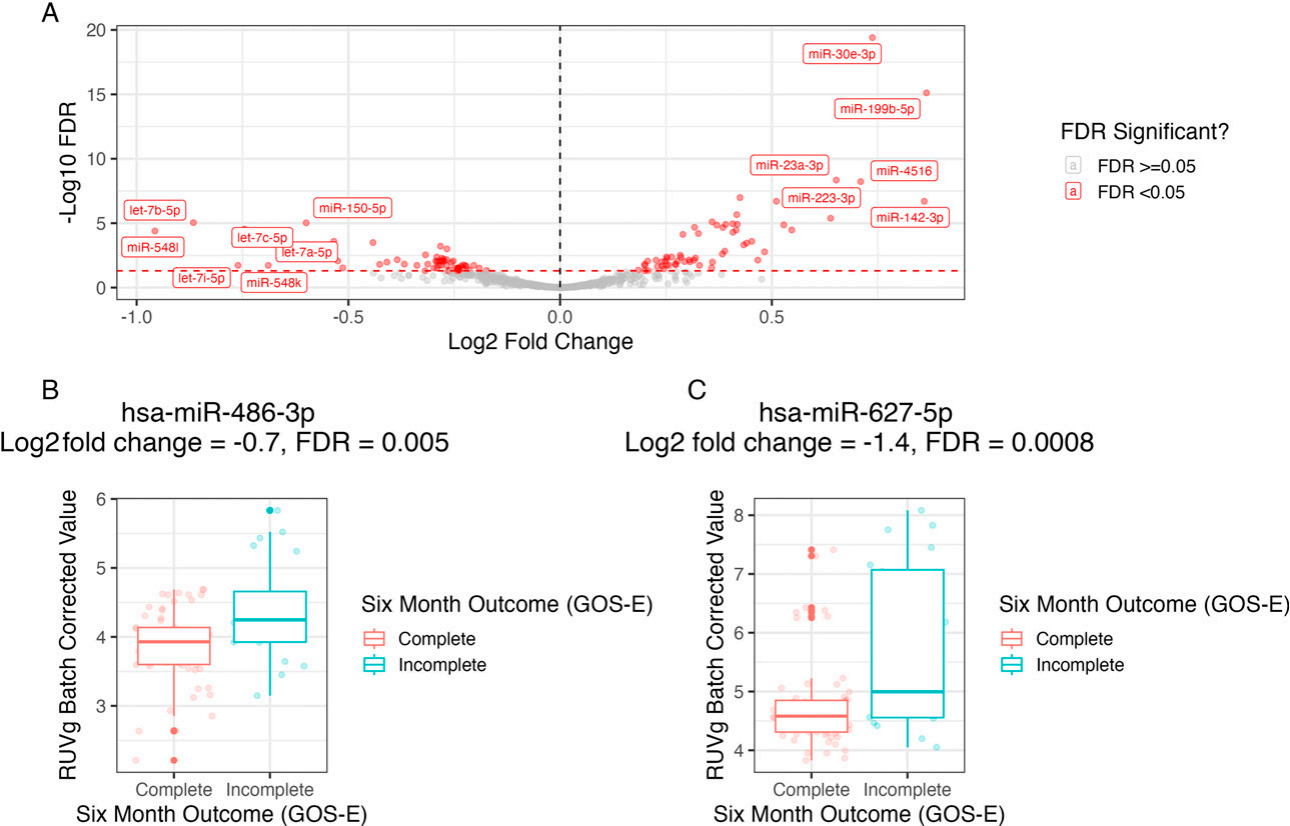
MicroRNAs (miRNAs) are differentially expressed in comparison of traumatic brain injury (TBI) with control and in association with Glasgow Outcome Scale-Extended (GOS-E) outcome at 6 months. Raw read data from control and day-1-post-injury samples were included in a DEseq2 model accounting for age, sex as a biological variable, self-reported race, and sequencing batch. miRNAs in which there was a fold-change >0.5 and a false discovery rate (FDR)-corrected *p* value <0.05 compared with control values were considered significant. Sixty-one miRNAs were upregulated, and 58 miRNAs were downregulated in TBI compared with control **(A)**. In a separate model, hsa-miR-486-3p **(B)** and hsa-miR-627-5p **(C)** were upregulated in participants with less favorable outcomes. GOS-E outcome was dichotomized as “complete recovery” (GOS-E = 8 at 6 months) compared with “incomplete recovery” (GOS-E < 8 at 6 months).

One hundred and nineteen miRNAs were altered in TBI participants compared with control individuals. Sixty-one (8%) miRNAs were differentially increased, while 58 (7%) miRNAs were decreased in those participants with TBI compared with control participants (Fig. 2A).

Eighty TBI participants (75%) had available 6-month GOS-E data (Table 1). Two miRNAs—hsa-miR-486-3p (log_2_ fold change = −0.7, FDR = 0.005) and hsa-miR-627-5p (log_2_ fold change = −1.4, FDR = 0.0008)—were upregulated within 24 h of injury in participants who had an incomplete recovery (GOS-E < 8) at 6 months after injury (Fig. 2B, C) compared with those who had complete recovery (GOS-E = 8) or control participants.

**FIG. 3.**
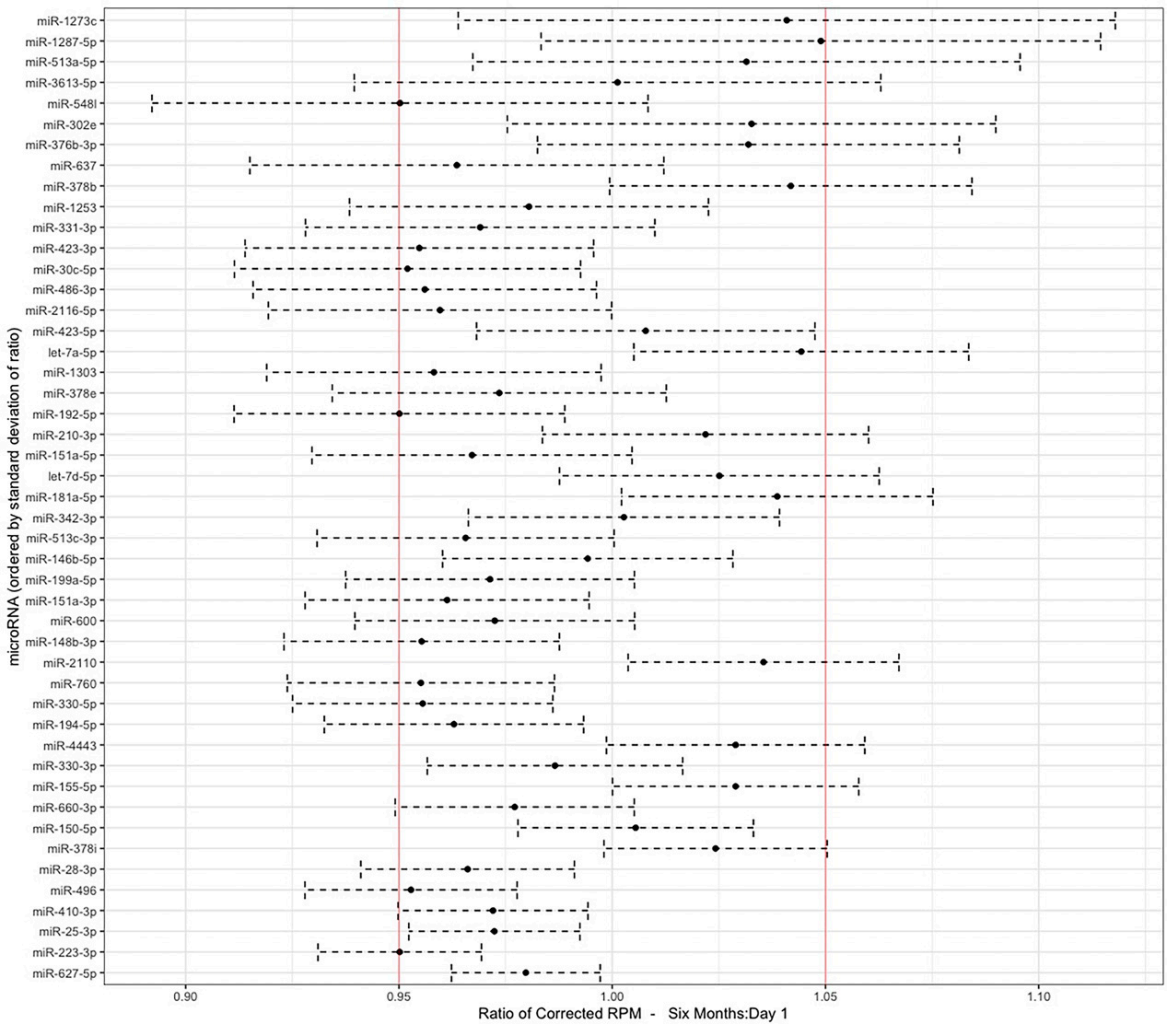
MicroRNAs (miRNAs) are persistently altered in whole blood at 6 months after injury. Twenty-three individuals with traumatic brain injury (TBI) had whole blood available at 6 months after injury. We calculated the ratio of RUVg batch-corrected miRNA expression at 6 months after injury compared with those from samples collected within 24 h of injury. We plot miRNAs with a mean ratio between 0.95 and 1.05, in ascending order by the standard deviation of the calculated ratio. Red lines represent a ratio of 0.95 and 1.05.

To examine the change in these differentially expressed miRNAs over time, we computed the ratio at 6 months postinjury to day 1 postinjury of the batch-corrected RUVg quantitation of differentially expressed miRNAs in *n* = 23 TBI participants who had samples both within 24 h of injury and at 6 months postinjury. A total of 50 of the 119 differentially expressed miRNAs had a mean ratio between 0.9 and 1.1 (Fig. 3), indicating that the level of these miRNAs was relatively stable over this 6-month time period in whole blood in this subset of TBI participants.

To determine whether batch effects were responsible for the differential expression of miRNAs, we next used quantitative PCR (qPCR). Three miRNAs with significant differential expression but varying mean read counts were chosen for further analysis. Hsa-let-7b-5p (base mean = 2867.7, log_2_ fold change = −0.9) was significantly decreased in TBI samples compared with control (2^-ΔΔCt^: 0.78 [0.74, 0.83]) and hsa-miR-223-3p (base mean = 48480.3, log_2_ fold change = 0.7) was significantly increased in TBI samples compared with control (2^-ΔΔCt^: 1.46 [1.36, 1.56]) (Supplementary Fig. S1). This was in accordance with the observed fold changes from differential expression analysis of the nCounter® data for hsa-let-7b-5p (log_2_ FC TBI vs. control = −0.94) and hsa-miR-223-3p (log_2_ FC TBI vs. control = 0.64). MiR-29a-3p (base mean = 71.3, log_2_ fold change = 0.46) was not significantly different by qPCR.

**FIG. 4.**
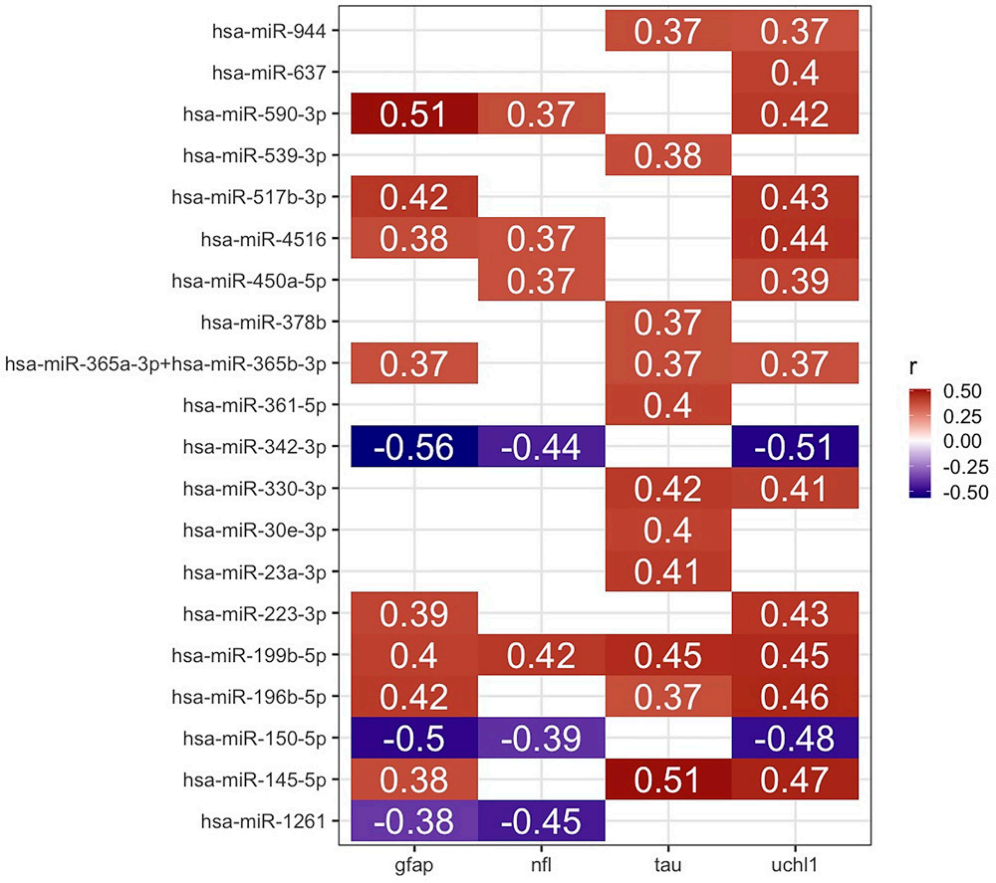
MicroRNAs (miRNAs) modestly correlate with brain-derived protein biomarkers. In *n* = 59 samples from traumatic brain injury (TBI) participants, concentrations of the protein biomarkers GFAP, UCHL1, tau, and NFL were correlated with levels of the *n* = 19 miRNAs that were differentially expressed in TBI participants compared with control participants. Boxes represent Spearman correlations with false discovery rate (FDR)-corrected *p* values <0.05. Brackets denote 95% confidence intervals. GFAP, glial fibrillary acidic protein; NFL, neurofilament light; UCHL1, ubiquitin C-terminal hydrolase-L1.

Finally, we report modest correlations between several differentially expressed miRNAs and the FDA-approved biomarkers GFAP and UCH-L1, as well as total tau and NFL (Fig. 4). Specifically, after Spearman correlation between all differentially expressed miRNAs and the four TBI protein biomarkers, *n* = 21 unique miRNAs significantly correlated with at least one of the four proteins after correction for multiple comparisons with the FDR. Four miRNAs (hsa-miR-551a, hsa-miR-342-3p, hsa-miR-150-5p, and hsa-miR-1261) were negatively correlated. The strongest correlations were observed between GFAP and hsa-miR-590-3p (Spearman’s ρ = 0.5, FDR = 0.004), total tau and hsa-miR-145-5p (Spearman’s ρ = 0.5, FDR = 0.004), GFAP and hsa-miR-342-3p (Spearman’s ρ = −0.6, FDR = 0.002), and UCHL1 and hsa-miR-342-3p (Spearman’s ρ = 0.5-, FDR = 0.004).

## Discussion

While TBI was originally conceptualized as a one-time injury followed by a period of extensive, and at times incomplete, recovery, it has more recently been designated as a chronic condition.^22,23^ The acute injury is followed by pathophysiological changes that may be maladaptive and exacerbate the insult, in a process known as secondary injury. Alternatively, adaptive responses promote healing and recovery. The cellular networks and regulatory pathways underlying these changes are poorly understood, leaving gaps in knowledge that have limited our ability to design targeted therapies. Thus, biomarkers that provide insight into these potential mechanisms are of great interest. The central role of miRNAs in regulating network protein expression makes them attractive candidate biomarkers that can lend further insights into disease pathophysiology.

In this study, we examined miRNAs in whole blood from participants who had sustained a recent TBI. Because miRNAs may inform the pathophysiologic mechanisms involved in the injury and/or recovery process, we explored miRNA alterations in the acute period following injury and associated these with neurological outcomes at 6 months. We identified two miRNAs (hsa-miR-486-3p and hsa-miR-627-5p) that were associated with clinical functional outcome as measured by the GOS-E at 6 months after injury.

Several studies have explored associations between miRNA and TBI. These studies vary with respect to the population studied, extent and severity of injury, miRNA assessment methodology, and biofluid source, including serum,^24,25^ plasma,^26,27^ and saliva.^28,29^ We selected whole blood for our experiments given that: (1) whole blood is more readily clinically assayed than serum or plasma preparations; (2) TBI can manifest with myriad systemic impacts; and (3) whole blood may provide a broader perspective on the systemic sequelae of brain trauma.

Of the 798 miRNAs tested, 119 were differentially expressed in TBI participants compared with controls. This number of miRNAs is consistent with similar studies in disease states.^30–32^ In a subset of participants for whom serial samples were available, these miRNAs were persistently altered 6 months after injury, suggesting that the acute perturbations may relate to long-term sequelae of injury.

We identified two miRNAs (hsa-miR-486-3p and hsa-miR-627-5p) that were associated with an unfavorable functional outcome, as determined by the GOS-E, at 6 months after injury. Given the mild nature of the majority of head injuries in our sample, we used a conservative definition of favorable outcome as complete recovery with no residual brain injury sequelae (GOS-E = 8). Yan et al.^33^ showed that miR-627-5p was upregulated in the serum of individuals with both mild and severe TBI. miR-627-5p is also increased in plasma exosomes in individuals who develop postoperative delirium after spine surgery.^34^ miR-486-3p exhibits altered expression in individuals with various cancers,^35^ including glioblastoma,^36^ and implicated with autism-spectrum disorders.^37^ miR-486-3p was found to be elevated in individuals with nontraumatic aneurysmal subarachnoid hemorrhage and correlated with a poor outcome 1 year after admission.^38,39^ However, little is known about the exact mechanisms of action of these miRNAs in the brain.

Our study also identified several other miRNAs that, while not correlated with GOS-E outcome, may provide additional insights into TBI pathophysiology. Prior studies have reported upregulation of miR-223-3p following sports concussion^40^ and mild TBI.^26^ Higher levels of plasma miR-223-3p on the day of concussion were associated with persistent symptoms 28 days postinjury.^41^ Other studies of sports-related concussion^40^ and mTBI^26^ have similarly found higher miR-223-3p levels following injury but this did not correlate with clinical outcomes. In a murine controlled cortical impact TBI model, miR-223-3p was upregulated in the peri-lesional tissue following TBI, resulting in neuronal dysfunction.^42^ Known targets of miR-223-3p include components of the NLRP3/IGF1 inflammasome pathway, which mediates cellular proliferation and migration and tissue hypertrophy. miR-186-5p, also identified in our study, impacts the NLRP3/IGF1 pathway in an *in vitro* TBI model.^43^ Exosomal miR-223-3p is increased in participants with small-vessel cerebrovascular disease-associated cognitive impairment compared with those with cerebrovascular disease alone or normal controls.^44^ Given that TBI is a known risk factor for late-life cognitive decline,^23,45,46^ investigating the role of miR-223-3p in response to TBI and small-vessel cerebrovascular disease may yield mechanistic targets for future therapies designed to mitigate these sequelae.

The miR-423 and miR-150 families have also been explored in several studies of central nervous system disease.^26,40^ Studies relying on targeted quantitative PCR of miR-423-3p, selected based on existing data or literature review, are among the most commonly assessed miRNAs in TBI.^26,41^ In contrast, miR-423-5p has been found to be dysregulated in glioma and brain metastases.^47,48^MiR-150-5p has been found in plasma extracellular vesicles in patients who have sustained TBI.^36^ Working hypotheses relate these elevations to regulation of bone remodeling following injury.^37^ Similar to miR-223-3p, miR-150-5p correlates with CSF biomarkers of Alzheimer’s disease and is differentially expressed in patient-derived peripheral blood mononuclear cells and postmortem brain tissue at autopsy.^36^ Future work to understand the blood compartments in which these miRNAs are distributed will elucidate their relation to the pathophysiology of injury.

In general, the miRNAs were only modestly correlated with protein biomarkers associated with head injury (Fig. 4), suggesting that these miRNAs may provide information that is orthogonal to what is available through currently available protein biomarkers. Prior data have shown that these protein biomarkers are prognostic of death and unfavorable outcome for those who present with GCS 3–12.^5,10^ Our study suggests that future work should evaluate if these miRNAs may be helpful in predicting outcome in those with less severe injuries and more favorable recovery trajectories, which represent the majority of TBIs.

Our study has several strengths. Relative to prior literature, we used a relatively large TBI cohort. The majority of prior studies examining miRNAs have examined smaller cohorts of fewer than 50 participants with TBI.^24,27,49–52^ Several of these studies have relied on targeted qPCR motivated by literature review.^24,26,50,51,53^ Our approach allowed an unbiased examination of miRNAs to uncover novel associations in the context of TBI and potentially allow a broader view of the alterations in circulating regulators following injury.

Our study has several important limitations. Given supply chain and recruitment barriers that arose during the COVID-19 pandemic, we performed miRNA analysis in three batches. For these reasons, we were neither able to prespecify a hypothesis nor perform power calculations. These practical considerations also limited our ability to standardize the timing of blood draw after injury beyond a stipulation to collect within 24 h of the index event. To mitigate these effects, we included sequencing batch in models of differential expression. We also used three distinct mathematical techniques to correct the input data for our regression models to account for any bias that these techniques may have introduced. While not statistically significant, our TBI participants are slightly older than our control participants. Furthermore, our participants were not matched on race, and our control group included participants with and without mild orthopedic injury. To improve the generalizability of our results, we included the demographic variables age, sex, and self-reported race in models of differential expression. Details of non-brain peripheral injuries, such as orthopedic fractures, were not collected at the time of injury in TBI participants.

Our study indicates that miRNAs are associated with TBI recovery when measured acutely after injury. Given the aforementioned limitations, further analysis in validation cohorts is necessary to determine whether this signal represents a generalizable prognostic biomarker. Furthermore, exploration of the cellular networks and pathways in which these miRNAs function, both in the brain and in other organ systems, may elucidate the mechanism underlying the observed association and motivate targeted mechanistic therapies.

### Transparency, rigor, and reproducibility summary

This study was not formally registered because it relied on a convenience sample from an ongoing biorepository. The analysis plan was not formally preregistered. A sample size of 100 subjects per group was planned based on the availability of biospecimens. Fluid biosamples were labeled using codes that were linked to participant identifying information but this was not available to investigators performing the biomarker measurements or data analysis. Samples were collected between 2018 and 2022. For miRNA analysis, whole blood samples were acquired directly into PAXgene RNA tubes within 24 h of injury. Tubes were inverted 8–10 times immediately following collection. Tubes were set at room temperature for 2 h, transferred to a −20°C freezer for 24 h, and then transferred to a −80°C freezer for prolonged storage. miRNA was isolated directly from the PAXgene tubes as described in the Methods section. miRNA was assayed in three different batches in the order of availability. The number of technical replicates for miRNA analysis was three per sample. Technical replicated results were combined by averaging values after masking outliers that were undetectable (read counts <20) before averaging. All equipment and analytical reagents used to perform measurements on the fluid biomarkers are widely available from commercial sources as listed in the article. The key inclusion criteria and clinical outcomes were assessed by investigators and their research staff with extensive expertise in clinical TBI research who had completed appropriate trainings. Statistical analyses were reviewed by Dr. Schneider, a faculty member in both biostatics/epidemiology and neurocritical care. Multiple comparisons were performed using FDR correction. Deidentified data from this study are not currently available in a public archive. Deidentified data from this study will be made available (as allowable according to IRB standards) by emailing the corresponding author at the time of publication. The analytic codes used to conduct these analyses presented in this study are not available in a public repository. They may be available by emailing the corresponding author at the time of publication. No future use of the Penn biofluid samples is possible because insufficient quantities remain. TRACK-TBI biosamples may be available and can be requested from the TRACK-TBI biorepository. The authors agree to provide the full content of the article on request by contacting the corresponding author.

## Abbreviations Used

CT: computed tomography
Ct: cycle thresholds
ED: emergency department
FDR: false discovery rate
GCS: Glasgow coma scale
GFAP: glial fibrillary acidic protein
GOS-E: Glasgow Coma Scale-Extended
miRNA: microRNA
mRNA: messenger RNA
MVA: motor vehicle accident
NFL: neurofilament light
PCA: principal components analysis
RUVg: remove unwanted variance method
TBI: traumatic brain injury
UCH-L1: ubiquitin C-terminal hydrolase-L1
