# Supplementary material for: MicroRNAs Detected in Whole Blood Following Traumatic Brain Injury Are Associated with Recovery 6 Months after Injury
Source: Neurotrauma Rep. 2025 Sep 26;6(1):903–14. doi: 10.1177/2689288X251380526 (PMC12547408; doi:10.1177/2689288X251380526)
Supplement: Supplementary Figure S1 [file 2689288x251380526_supplementary_figure_s1.docx]

**
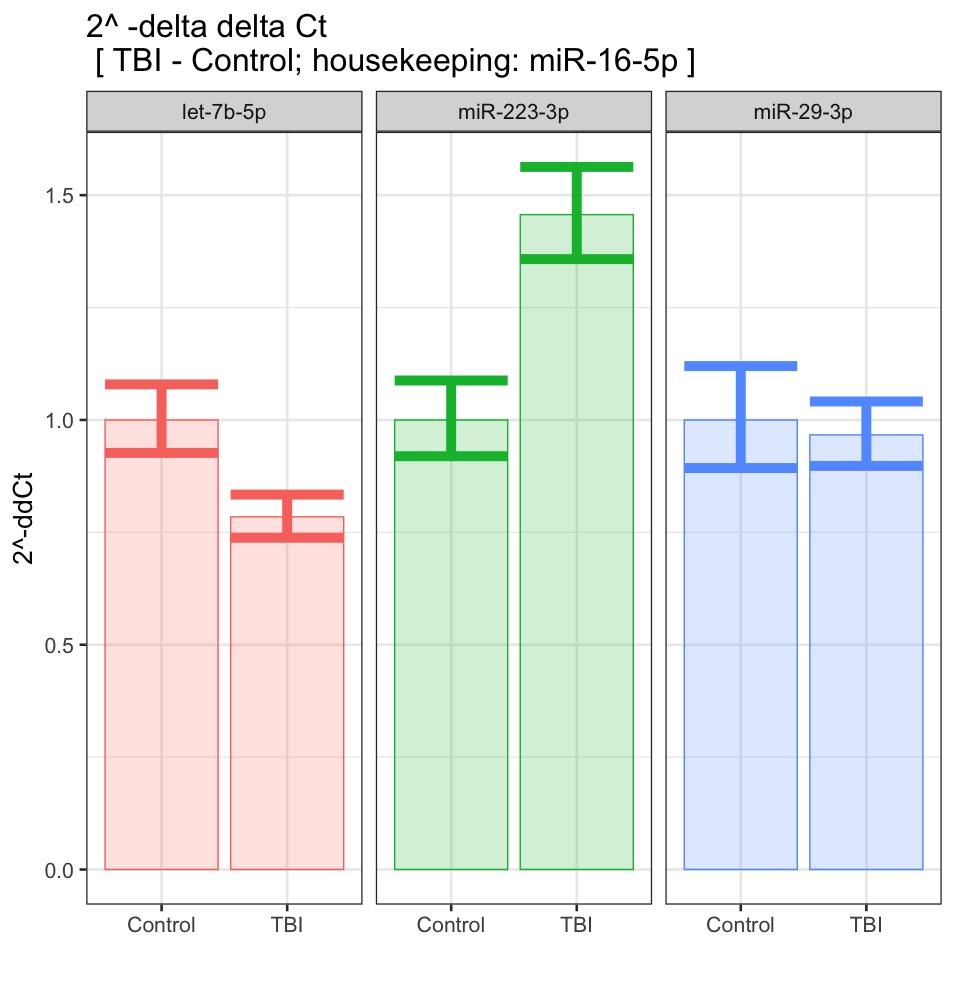
**

**Supplemental Figure 1. Quantitative PCR of select miRNAs**. We performed the ThermoFisher TaqMan miRNA qPCR assays against four miRNAs: let-7b-5p, miR-223-3p, miR-29-3p, and miR-16-5p. We then performed qPCR in 16 TBI and 16 control samples. We present boxplots of the exponentiated delta delta Ct values, calculated by first normalizing to sample specific miR-16-5p as a housekeeping miRNA, then calculating the mean, and finally subtracting the mean in TBI patients from the mean in control patients. Let-7b-5p is significantly decreased among TBI samples and miR-223-3p is significantly increased among TBI samples, in accordance with Nanostring data.
